# Mr. Rolfe's Cases of Dropsy, &c.

**Published:** 1801-05

**Authors:** W. D. Rolfe

**Affiliations:** Dispensary House, North Street


### *To the Editors of the Medical and Physical Journal.*

GENTLEMEN,

THE following are the Cafes I promifed in the laft Number:

JAMES RICE, aged 18, was admitted the 31ft of May, 1800; he had great fever, cough troublefome, breath very fhort; I gave him, viz. ℞. pulv. ipecac. ℈j. s. s. oxy. fcillæ, ℥j. capt. coch. j. parv. fæpe urgenti tuffe, infus. amar. ℔. j. capt. poculo bis die. I was taken ill myfelf this day, therefore faw him no more for feveral weeks, as my bufinefs was turned over to another perfon until I was well; but, upon ftrict enquiry afterwards, I found he began to fwell from that time in his legs, abdomen, &c. with great pain, and little or no urine; every fymptom feemed gradually to increafe, until he was fuppofed to be in a dying ftate, therefore the medical attendance was defifted from until the 25th of July, when his mother applied to me again. I found him with a general dropfy, and very much emaciated; the ftomach, abdomen, and lower extremities extremely diftended; the penis and fcrotum in proportion; could reft only in an erect pofition, making only a tea cup full of turbid urine in twenty-four hours. I then gave him, ℞. Pulv. fol. digital. g. j. pulv. aromat. g. iv. pulv. glycirrh. g. xv. ft. pulv. capt. j. ter die. 26th. I found a diarrhoea had came on in the morning, and he felt himfelf extremely weak; I gave him opii. g. jfs. and punctured the penis and fcrotum. 28th. Symptoms nearly the fame; rept. pulv. digital. ut antea. Aug. 1ft. The diarrhoea ftill continues; had fome intervals of fleep, makes rather more urine, the penis and fcrotum quite evacuated; Rept. pulv. digital. cum opio. j. ¼, in fing. ch. 2d. Has had fome fleep in the night, and much freer from pain; the diarrhoea moderated, makes a pint of urine in twenty-four hours; Rept. pulv. digital. e opii ut antea. 5th. Much eafier, makes more urine, the cough troublefome; Rept. pulv. ut ultimo. 8th. Diarrhoea abated, cough lefs, the fwellings generally abated, makes two pints of urine in twenty-four hours; Rept. pulv. ut ultimo. 11th. Every fymptom much abated, urine plentiful, was able to lie down and have fome refrefhing fleep, walked out a little; Rept. pulv. ut ultimo et poculo decoct. cinchonæ bis die. 15th. Continued much better, left off his powders gradually, and continued the decoct. cinchonæ until the 18th of Auguft, when he was difcharged perfectly cured.

ELIZ. REYNOLDS, aged 50, was admitted Auguft 15, 1800, with with anafarca and afcites, and had been long ill with cough and other hectic fymptoms, alfo very much in pain; made very little urine; breath very fhort. I gave her pulv. digital. e opii ut (Rice) ter die. 16th. Nearly the fame. 18th. Rather more urine; Rept. pulv. ut antea. 19th. Ditto. 20th. Very much in pain; ℞. Gamb. g. iij. crem. tart. ʒj. M. ft. et divid. in dofæ ij. capt. j. ftatim et rept. h. s. si. opus fuerit. 21ft. Both powders were taken, but produced no effect; vomited water at times; ℞. Pulv. fcillæ g. vj. opii g. iij. cons. q. s. ft. pil. No. vj. capt. j. 4tis horis. 22d. Had taken all the pills, but no effect; pain and ficknefs ftill continue, rather more urine, the abdomen, &c. very much diftended, breath very fhort; gave her infus. digital. ℥viifs. fpirit. æth. nitrofæ ℥fs. tinct. opii gt. 60, ft. mift. capt. coch. ij. ampla 4tis horis. 23d. Every fymptom the fame. I performed paracentefis, and drawed off twenty-three pints of water, and fhe died the next day.

Sarah Richards, aged 36, was admitted October 16, 1801, with anafarca and afcites, and general marks of debility, and had lately lain-in; urine very fcanty. I gave her, ℞. Pulv. digital. gr. j. pulv. aromat. g. iv. pulv. glycerrh. g. xv. ft. pulv. ter die fum. 18th. Nearly the fame; Rept. pulv. ut antea. 20th. Ditto; ℞. pulv. fcillæ calomel pp. aa. g. vi. cons. q. s. ft. pil. No. iv. h. s. s. 22d. Had fome watery ftools; fwellings rather loofened, and makes rather more urine. 25. Legs lefs fwelled, and makes more urine. 27. Ditto. 31. Ditto. Rep. pil. calomel et fcillæ h. s. s. Nov. 1. Continues mending; had feveral watery ftools, and made more urine; fwelling confiderably decreafed. 3. A diarrhœa came on; fwelling of the legs much lefs, urine plentiful. Repet. pulv. ut antea bis die, et poculo decoct. cinchonæ bis die. 6. Swelling nearly gone. Rep. pulv. et decoct. 12. Continued mending, and the 20th was difcharged cured.

Esther Shalswell, aged 55, was admitted October 23, 1800, with anafarca; legs much fwelled, and in pain, with fome degree of inflammation. I gave her ℞. Pulv. digital. &c. ut (Richards). 24th. Nearly the fame; Rept. pulv. ut antea. 27th. The fkin rather loofer; made rather more urine. 29th. A diarrhœa came on, and made more urine; Rept. pulv. ut antea. 31ft. Swelling much lefs, makes plentiful urine. Nov. 1ft. Ditto. 3d. Ditto. 12th. Swelling quite gone; gave her decoct. cinchonæ, and was difcharged the 15th cured.

William Turner, aged 84, was admitted July 26, 1800, with hydrothorax, &c. Of a fpare habit of body, had not lain down in bed for above feven years, always fat perfectly erect in his chair; legs much fwelled, breath fhort, dry cough, urine very fcanty; gave him Pulv. digital, &c. ut (Richards) ter die.

28th.

28th. Rather more urine. 30th. Ditto. Auguft 1ft. Rather coftive; ℞. Pulv. crem. tart. g. x. pulv. jalap ℈j. ft. pulv s. s. 4th. More open, and rather more urine; Rept. pulv. digital. ut ult. 5th. Symptoms nearly the fame; Rept. pulv. cath. ut antea. 6th. A diarrhœa came on in the evening; he went up ftairs to call his wife, fat himfelf down on the foot of the bed at eleven o'clock, fell faft afleep, and fell backwards on the bed, and flept quietly until three the next morning, when he awoke frightened. 8th. Continued open, made confiderable quantity of water; Rept. pulv. ut antea. 12th. Diarrhœa continued very troublefome, but made plentiful urine; ℞. Pulv. creta. comp. e opii ℈j. ft. pulv. ter die. 14th. Swelling much lefs, griping troublefome; ℞. Pulv. rhab. ℈j. ft. pulv. s. s. Rept. pulv. digital. ut antea; urine plentiful. 18th. Swelling much lefs, cough and other fymptoms much abated; Rept. pulv. rhab. et pulv. digital. ut antea. He went to bed regularly, and flept well. 22d. Pain and griping troublefome, gave him ℞. opii crud. g. ij. acid. nitros ʒij. aq. font. ℥ij. ft. gutt. capt. coch. j. parv. 4tis horis. 27th. Every fymptom much abated, urine plentiful; Rept. pulv. digital. ut antea cum infus. amar. until the 1ft of September, when he was difcharged cured.

Ann Rice, (mother to James Rice) aged 56, was admitted Jan. 3, 1801, with anafarca and afcites; makes a pint of water in twenty-four hours; dry cough, breath fhort. ℞. Pil. fcillæ cum calomel et pulv. digital. &c. ut (Richards). 5. Had fome watery ftools; other fymptoms nearly the fame. ℞. Pulv. jalap ℈j. crem tart. g. x. ft. pulv. s. s. 6. Nearly the fame. Rep. pulv. digital ut antea. 9. Ditto. 12. Swelling much lefs, urine plentiful. Rep. pulv. digital. ut antea et decoct. cinch. bis die. 20. Continued the digital. et cinch. until the 30th, and was difcharged cured.

Hannah Morress, aged 46, was admitted Jan. 20, 1801, with afcites, the abdomen much fwelled, and greatly in pain; urine fcanty. ℞. Pil. fcillæ et calomel ut (Richards) et pulv. digital. &c. ter die. 22. Had feveral loofe ftools, made rather more urine. Rep. pulv. digital. et infus. amar. 24. Urine more plentiful, cough troublefome. 27. Symptoms much abated. Rep. pulv. et infus. alfo oxy. fcillæ capt. coch. j. min. fæpe urgenti tuffe. Feb. 3. Every fymptom much abated; urine plentiful. Continued the digital. et infus. amar. until the 20th, and was difcharged cured.

John Williams, aged 58, was admitted Jan. 23, 1801, with cough, difpnœa, and fwelling of the abdomen; urine fcanty, and much fever. ℞. Pulv. jalap ℈j. crem. tart. gt. x. ft. pulv. s. s. oxy. fcil. ℥j. cap. coch. min. fæpe, pulv. feb. nitrofæ, trosæ, 4tis horis. 24th. Had one stool, cough still troublesome, legs swelled, breath very short, urine scanty. ℞. Pil. scillæ et calomel ut (Richards); rep. oxy. scillæ et pulv. feb. nitrosæ. 26. Symptoms nearly the same; rep. pil. scillæ et calomel. 31. Had some watery stools; breath rather better, fever rather less, cough at night troublesome. ℞. Pil. sapon. g. x. omni nocte sum. pulv. digital. et infus. amar. (Richards). Feb. 3. Cough continues troublesome. Rep. pulv. digital. &c. et infus. amar. Swelling of the abdomen still continues, urine scanty. 7. Increased the digitalis to four grains a day. 9, 12, Took digitalis six grains a day until the 22d, with relief, when I gave him Crem. tart. mellis aa. ℥ijss. ft. elect. cap. 3tiam partem omni die. He continued the use of this electuary with tonics until March 30, when he was discharged cured.

Sarah Holliday, aged 21, was admitted Jan. 24, 1801, with general dropsy; had been out-patient to an hospital for several weeks, but without relief; has been in this state three months; makes only three-quarters of a pint of water in 24 hours; obliged to sit perfectly erect; has not lain down in bed for some time; her legs greatly swelled. ℞. Pil. scillæ et calomel ut (Richards) et pulv. digital. &c. ter die. 27. Has had two stools; swelling rather less in her arms; has made rather more urine. Rep. pulv. digital. ut antea. 31. Nearly the same. Rep. pulv. digital. &c. et infus. amar. Feb. 3. Ditto. Rep. pulv. ut antea. 6th. Legs more swelled; punctured the legs and gave her decoct. cinchonæ. 14th. Legs no less; increased the digitalis until the 20th without effect, but found her much worse, and supposed almost in a dying state. I then gave her, ℞. Crem. tart. mellis aa. ℥iss. ft. elect. ut antea sum. 22d. Found her rather easier, a little more urine; Rept. elect. ut antea, capt. dimid. o. die. 24th. Found her much easier, swellings much less, makes more urine. 26th. Still better; Rept. elect. diuret. ut antea. The urine continued to come freely; she continued the electuary and decoct. cinchonæ until March 28, when she was discharged cured.

William Davis, aged 58, was admitted Jan. 24, 1801; had been ill for two months with peripneumonia, and violent pain in the epigastric region; and now the abdomen began to swell. ℞. Pil. calomel et scillæ ut (Richards), et pulv. digital. &c. bis die. 26th. Urine scanty, and much in pain; had two loose stools; Rept. pulv. digital. &c. ter die, and crem. tart. dissolved in boiling water for common drink. 29th. Nearly the same; Rept. pulv. digital. ut antea 4tis de die. 31. Swelling still continues, with much sickness; ℞. Infus. digital. ℥viiss. spirit. æth. nitrosæ ℥ss. ft. mist. capt. coch. iij. ampt. 4tis horis. Feb. 2d. Urine small, pain continues, sickness continues; Rept. mist. ut antea. 3d. Nearly the same; Rept. mist. adde conf. arom. ℥ij. 4th. Sickness rather abated. 5th. Nearly the same; Rept. mist. ut ultimo. 6th. He died.

John Horner, aged 58, was admitted Jan. 30, 1801, with general dropsy; had been a patient in the Hospital thirteen weeks without relief; had not lain down in his bed for three weeks, was obliged to sit perfectly erect, breath extremely short, makes one pint of urine in twenty-four hours; ℞ Pil. calomel et scillæ ut (Richards) et pulv. digital. &c. bis die. 31st. Had one costive stool; urine the same. Rept. pil. Feb. 2d. Very much in pain; Rept. pil. ut antea. 4th. Had two costive stools, urine the same, pain the same; ℞. Pulv. jalap ℈j. pulv. crem. tart. g. x. ft. pulv. s. s. Rept. pulv. ut antea ter die. 5th. Nearly the same; ℞. Infus. digital. ℥viiss. spirit. æth. nitrosæ ℥ss. ft. mist. capt. coch. iij. amp. 4tis horis. 6th He died.

William Mann, aged 32, was admitted Dec. 9, 1800; had been ill a long time with phthisis pulmonalis. Took tinct. digitalis et infus. amar. and was discharged, Jan. 22d, cured. Has since been ill again, with cough, breath short, and great expectoration of purulent matter; and still remains the same under my care.

Richard Batting, aged 60, was admitted Dec. 9, 1800, with asthma, dry cough, and breath extremely short. Took tinct. digitalis cum infus. amar. by increased doses, until Jan. 2, when he was discharged cured.

Sarah Raxworthy, aged 55, was admitted Jan. 9, 1801, with long standing asthma, breath short, cough troublesome; took tinct. digital. et infus. amar. by increased doses for six weeks, then placed under another person's care, and died soon afterwards.

William Shepherd, aged 80, was admitted Jan. 1, 1801, with long continued asthma, but now unable to lie down in bed, with every symptom of hydrothorax and anasarca beginning; took tinct. digital. et infus. amar. and was discharged, Feb. 14, cured. Was admitted again a month afterwards, and died suddenly.

Griffiths Loyd, aged 75, was admitted Jan. 17, 1801; has had asthma for many years; his legs now begin to swell, and urine scanty; took tinct. digital. by increased doses, and was discharged, March 2, cured.

Ann Ramsey, aged 40, was admitted Jan. 27, 1801, with consumption; no rest at night, and continued diarrhœa. ℞. Opii. g. j. o. n. et tinct. digital. She died Feb. 28.

Mary Melsome, aged 53, was admitted Feb. 3, 1801; has had asthma for twenty years; has been worse two weeks; legs legs ſwell, urine ſcanty; took tinct. digital. at increaſed doſes, and was diſcharged, March 20, greatly relieved.

HESTER BROWNING, aged 50, admitted Feb. 3, 1801; has had aſthma for three years, breath now extremely ſhort, expectoration much, legs much ſwelled, makes ℔ſs. of water in twenty-four hours. Took digitalis by increaſed doſes for a month without relief; I then gave her, ℞. Crem. tart. mellis aa. ℥iſs. ft. elect. which ſhe continued until the 24th of March, when ſhe was diſcharged cured.

A great many other caſes of the like kind I could inſert, if my time would permit; but I hope this will be ſufficient to ſhow that Digitalis cannot, *at all times*, be depended on. I here meant only to ſtate the clear caſes under my own immediate attention; I do not preſume, as a junior practitioner, to throw any new light on practice or diſeaſes, but merely to ſtate caſes, and leave my elders to judge of them.

*Diſpenſary Houſe, North Street,*

